# PLS3 Overexpression Delays Ataxia in *Chp1* Mutant Mice

**DOI:** 10.3389/fnins.2019.00993

**Published:** 2019-09-19

**Authors:** Eva Janzen, Lisa Wolff, Natalia Mendoza-Ferreira, Kristina Hupperich, Andrea Delle Vedove, Seyyedmohsen Hosseinibarkooie, Min Jeong Kye, Brunhilde Wirth

**Affiliations:** ^1^Institute of Human Genetics, Center for Molecular Medicine Cologne, Institute for Genetics, University of Cologne, Cologne, Germany; ^2^Center for Rare Diseases Cologne, Institute for Genetics, University of Cologne, Cologne, Germany

**Keywords:** plastin 3 (PLS3), calcineurin like EF-hand protein 1 (CHP1), Na^+^/H^+^ exchanger (NHE1), ataxia, neurodegeneration, modifier

## Abstract

Many neurodegenerative disorders share common pathogenic pathways such as endocytic defects, Ca^2+^ misregulation and defects in actin dynamics. Factors acting on these shared pathways are highly interesting as a therapeutic target. Plastin 3 (PLS3), a proven protective modifier of spinal muscular atrophy across species, is a remarkable example of the former, and thereby offers high potential as a cross-disease modifier. Importantly, PLS3 has been linked to numerous proteins associated with various neurodegenerative diseases. Among them, PLS3 directly interacts with calcineurin like EF-hand protein 1 (CHP1), whose loss-of-function results in ataxia. In this study, we aimed to determine whether PLS3 is a cross-disease modifier for ataxia caused by *Chp1* mutation in mice. For this purpose, we generated *Chp1* mutant mice, named *vacillator* mice, overexpressing a *PLS3* transgene. Here, we show that PLS3 overexpression (OE) delays the ataxic phenotype of the *vacillator* mice at an early but not later disease stage. Furthermore, we demonstrated that PLS3 OE ameliorates axon hypertrophy and axonal swellings in Purkinje neurons thereby slowing down neurodegeneration. Mechanistically, we found that PLS3 OE in the cerebellum shows a trend of increased membrane targeting and/or expression of Na^+^/H^+^ exchanger (NHE1), an important CHP1 binding partner and a causative gene for ataxia, when mutated in humans and mice. This data supports the hypothesis that PLS3 is a cross-disease genetic modifier for CHP1-causing ataxia and spinal muscular atrophy.

## Introduction

Cerebellar ataxias are a group of neurodegenerative disorders mainly characterized by progressive cerebellar degeneration leading to movement incoordination and unsteadiness. Autosomal recessive cerebellar ataxias (ARCAs) account for the most heterogeneous group of hereditary ataxias, whose onset occurs generally before 20 years of age. To date, a broad range of ARCA-causing genes have been identified, being involved in various pathways including (1) DNA repair, (2) mitochondria, (3) metabolism, including lipoprotein assembly, and (4) chaperone-mediated protein folding and aggregation (Fogel and Perlman, 2007; Anheim et al., 2012). Additional disease-causing mutations have been reported in genes involved in cytoskeleton, vesicular trafficking, synaptic transmission, calcium homoeostasis and pH regulation (Anheim et al., 2012; Thomas et al., 2014; Guissart et al., 2015; Mendoza-Ferreira et al., 2018).

Recently, calcineurin like EF hand protein 1 (*CHP1*) has been identified as an ARCA-causing gene in humans and mice. Mechanistically, diminished levels of CHP1 resulted in defective NHE1 glycosylation and membrane translocation to axon terminals, causing a disturbed proton homeostasis eventually resulting in Purkinje neuron degeneration (Liu et al., 2013; Mendoza-Ferreira et al., 2018).

In a previous study, we showed that Plastin 3 (PLS3) is a Ca^2+^-independent interacting partner of CHP1 (Janzen et al., 2018). PLS3 is a broadly expressed F-actin-binding and -bundling protein essential for F-actin stabilization (Lin et al., 1994; Giganti et al., 2005). PLS3 overexpression (OE) has been extensively demonstrated to be a protective modifier for the devastating neurodegenerative disorder spinal muscular atrophy (SMA) in humans and numerous SMA models (Oprea et al., 2008; Dimitriadi et al., 2010; Hao Le et al., 2012; Ackermann et al., 2013; Hosseinibarkooie et al., 2016; Kaifer et al., 2017). Further analysis of the PLS3 interactome uncovered several putative PLS3 interacting partners, which are associated with various neurodegenerative diseases, such as amyotrophic lateral sclerosis (ALS), Charcot-Marie-Tooth disease (CMT), and ARCA (Hosseinibarkooie, 2016). Importantly, many neurodegenerative disorders share common pathogenic pathways, including endocytic defects and Ca^2+^ misregulation as well as disturbed actin dynamics (Hosseinibarkooie et al., 2016, 2017; Schreij et al., 2016; Riessland et al., 2017; Hensel and Claus, 2018). PLS3 OE has been demonstrated to improve these shared disease pathways in SMA (Oprea et al., 2008; Hosseinibarkooie et al., 2016). Thus, we speculated that PLS3 OE might be a cross-disease modifier, targeting common neuropathogenic pathways.

Since, it has been shown that PLS3 directly interacts with CHP1, and CHP1 depletion causes ataxia in the *vacillator* mouse model, we hypothesized that PLS3 OE may improve the ataxic phenotype *in vivo*. For that purpose, we crossbred *Chp1* mutant *vacillator* mice, which carry biallelic splice-site variants in *Chp1* that dramatically reduce, CHP1 levels (Liu et al., 2013) with the ubiquitously *PLS3* overexpressing transgenic mice (Ackermann et al., 2013). Importantly, we demonstrate in this study that PLS3 OE, does not fully rescue the disease phenotype, but delays the ataxic phenotype at an early disease stage by preventing axon degeneration of Purkinje neuron. Moreover, we show that, PLS3 OE in the cerebellum shows a trend of increased membrane targeting and/or expression of Na^+^/H^+^ exchanger (NHE1). In conclusion, this study reveals that PLS3 OE is a disease modifier for ataxia caused by *Chp1*-depletion.

## Materials and Methods

### Mouse Experiments

#### Mouse Model

**FIGURE 1:**
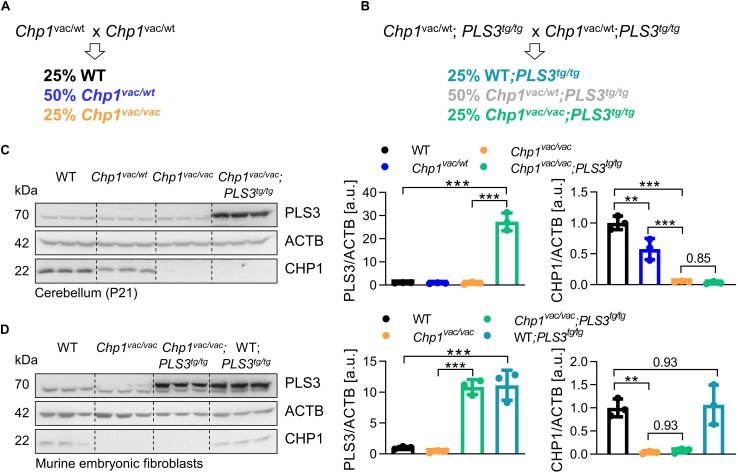
Generation of PLS3 OE *vacillator* (*Chp1^vac/vac^;PLS3^tg/tg^*) mice. **(A,B)** Breeding strategies to generate WT and *Chp1*^vac/vac^ mice and *Chp1^vac/vac^;PLS3^tg/tg^* mice in a congenic C57BL/6N background. **(C,D)** Western blot analysis and quantification of PLS3 and CHP1 levels confirmed the overexpression of *PLS3V5* in cerebellum at 3-weeks of age and in murine embryonic fibroblasts (MEFs) from *Chp1^vac/vac^;PLS3^tg/tg^* and *PLS3*^tg/tg^ mice (*N* = 3). CHP1 levels were not altered upon PLS3 OE. PLS3 staining: upper band = transgenic PLS3V5, lower band = endogenous PLS3. ACTB: loading control. ^∗∗^*p* < 0.01, ^∗∗∗^*p* < 0.001, one-way ANOVA and Holm–Sidak’s multiple comparisons test. Error bars represent SD.

All mouse experiments were approved by LANUV NRW (reference number: 84-02.04.2014.A242). The *vacillator* mouse model (B6.Cg-CHP1vac) (Liu et al., 2013) was provided by Susan L. Ackerman, Howard Hughes Medical Institute and Jackson Laboratory, Bar Harbor, ME, United States. All mouse lines were backcrossed for at least seven generations to obtain a congenic C57BL/6N background. In order to overexpress PLS3 in the *vacillator* mouse model, *Chp1*^vac/vac^ females were crossed with *PLS3*^tg/tg^ males. The heterozygous offspring were backcrossed with *PLS3*^tg/tg^ mice to obtain homozygous *PLS3* OE. The *PLS3* transgene harbors a V5 tag (Ackermann et al., 2013). To generate all required genotypes, we followed the depicted breeding strategies (Figures 1A,B). Genotyping for *Chp1* and *PLS3V5* was performed as previously described (Ackermann et al., 2013; Liu et al., 2013).

#### Beam-Walking Assay and Footprint Pattern Analysis

The ataxia phenotype was assessed using the beam-walking assay as previously described (Stanley et al., 2005). Prior to the test situation, mice were trained for a short time period to walk along a ruler (60 cm long, 3 cm wide, and 30 cm elevated) toward a goal box. Next, three trials were performed for each mouse. For this, mice were placed on the beam (50 cm long, 8 mm diameter, and 30 cm elevated) and allowed to walk toward the goal box. When a mouse fell from the beam, the time was stopped and the mouse was returned to the position it fell from. Motor coordination performance was assessed by measuring the time the mouse needed to cross the beam. The test was first performed with 3-weeks-old mice and repeated at 4-weeks of age, subsequently the test was performed every second week until 12-weeks of age. Mice developing ataxia were not able to perform the test after 6-weeks of age.

For footprint pattern analysis, the rear paws of the mouse were colored with nontoxic black finger paint whereas the front paws were colored in red. Next, the mouse walked through a tunnel (40 cm long, 7 cm wide and 5 cm high) over a white paper. For quantification, the mean hind feet, stride length and stride width values were determined for each mouse by measuring 3 steps per animal (Tsai et al., 2012). The front/hind feet overlap was analyzed by measuring the distance (in cm) between the center of the left or right front paw footprint to the center of the following hind paw footprint. A complete overlap, meaning the center of the hind footprint fell on top of the front footprint, equals zero. 4–12 animals per genotype were included of which 3–10 steps were assessed (Carter et al., 1999).

### Primary Murine Embryonic Fibroblast Cultures

Primary murine embryonic fibroblasts (MEFs) were isolated from E12.5 to 13.5 animals as previously described (Ackermann et al., 2013). Cells were grown under standard cell culture conditions (37°C, 5% CO_2_; DMEM, 10% FCS, 10 U/ml Pen/Strep, 250 μg/ml Amphotericin B).

### Immunostainings and Western Blotting

To measure protein levels, Western blotting (WB) was performed according to standard protocols. For analysis, the following primary antibodies were used; anti-CHP1 (Thermo Fisher Scientific; PA5-29876), anti-NHE1 (SCBT; 4E9), anti-PLS3 [Eurogentec (Oprea et al., 2008); 1238], anti-ACTB (Sigma, A5316), anti-GAPDH (Sigma, G-9295), anti-HSP90 (Cell Signaling; 4877), anti-EGFR (SCBT; sc-03), and anti-pan-cadherin (Abcam; ab6528).

### Subcellular Protein Fractionation

For subcellular fractionation, the Subcellular Protein Fractionation Kit for Tissues (Thermo Fisher Scientific) was used. We followed the manufacturer’s instruction.

### Histology

For histological analysis of the cerebellum, anesthetized mice (ketamine/xylazine: 0.1 ml/10 g) were fixed by transcardial perfusion using 4% PFA. For further fixation, brains were incubated overnight in 4% PFA at 4°C and processed as described in standard protocols for cryo (15 μm sections) and paraffin sectioning (5 μm sections). For immunofluorescent staining of cryo-sections, following primary antibodies were used: anti-calbindin-D28k (Swant; CB300), anti-PKCγ (Abcam; ab71558). Calbindin-D28k immunohistological stainings were performed as described in Storbeck et al. (2014) using an ABC kit (Vector Laboratories). To acquire the whole Purkinje neuron cell body, different number of stacks were included in the analysis. All samples were imaged simultaneously with the same settings. The experimentator was blinded at all steps to exclude any bias during imaging.

### Microscope

All images were acquired using either the fully motorized fluorescent microscope AxioImager.M2, equipped with an AxioCam MRm camera and an ApoTome2 device mimicking confocality (Zeiss), or the bright field microscope Axioskop2 with an AxioCam ICc1 camera (Zeiss). Fluorescent images were acquired as Z-stacks. For calbindin (CAB)-positive area quantification, 5–10 stacks (stack: 0.5 μm thick) were acquired in order to image complete Purkinje neuron cell bodies. The CAB-positive area was analyzed in an unbiased approach using a Macro in Fiji. Using an automatically set fluorescence intensity threshold and a fixed size region of interest, the macro measured the total CAB-positive area per image in the granule cell layer in lobes I-II of the cerebellum. 6–12 images per mouse were used for quantification. Since early Purkinje neuron loss was detected in lobe I-II, all quantifications were performed in this region.

### Statistical Analysis

All experiments were performed in a double-blinded manner. Statistical analyses were performed using the software GraphPad Prism7. Prior to statistical analysis, we performed a distribution analysis applying the Shapiro–Wilk test. For all normally distributed data a one-way ANOVA and Holm–Sidak’s *post hoc* test for multiple comparisons was applied to evaluate the statistical significance between multiple groups. All data are represented as mean ± SD. Three levels of statistical significance were considered: ^∗^*p* < 0.05, ^∗∗^*p* < 0.01, and ^∗∗∗^*p* < 0.001.

## Results

### Generation of PLS3 OE *Vacillator* Mice

In order to test our hypothesis whether PLS3 OE is a cross-disease modifier ameliorating the ataxic phenotype caused by *Chp1* depletion *in vivo*, we crossbred *vacillator* mice with transgenic homozygous PLS3 OE mice, where the *PLS3* transgene is driven under the control of the ubiquitously expressing CMV promoter and has a V5 tag (Ackermann et al., 2013). To obtain the desired genotypes for this study, we followed the depicted breeding strategies (Figures 1A,B). Important to mention, all *PLS3* transgenic mice overexpress the transgene homozygously. To confirm PLS3 OE in *vacillator* mice, we analyzed PLS3V5 levels in cerebellum and primary MEFs lysates by WB. Indeed, PLS3V5 levels were 27-fold increased in cerebellum (Figure 1C) and 11-fold in MEFs (Figure 1D) of *Chp1^vac/vac^;PLS3^tg/tg^* mice. Noteworthy, PLS3 OE did neither alter CHP1 levels in cerebellum nor in MEFs (Figures 1C,D). These results confirm that we successfully generated *Chp1* mutant *vacillator* mice overexpressing human PLS3.

### PLS3 OE Ameliorates the Ataxic Phenotype in *Vacillator* Mice at an Early Disease Stage

Since ataxia is defined by the lack of motoric coordination of voluntary muscles and the concomitant gait abnormalities, we first investigated the effect of PLS3 OE on the motoric phenotype. In order to analyze the disease progression over time, we performed two coordination/motoric tests: first, the beam-walking motor-balancing and coordination test at 3, 4, and 6 weeks, and second, the footprint pattern analysis at 3-, 4-, 6-, 8-, and 12-weeks of age. Notably, at 8 weeks of age, *Chp1*^vac/vac^ and *Chp1^vac/vac^;PLS3^tg/tg^* mice were no longer able to perform the beam-walking test due to strong balance and coordination deficits, therefore, we were unable to perform further analysis.

**FIGURE 2:**
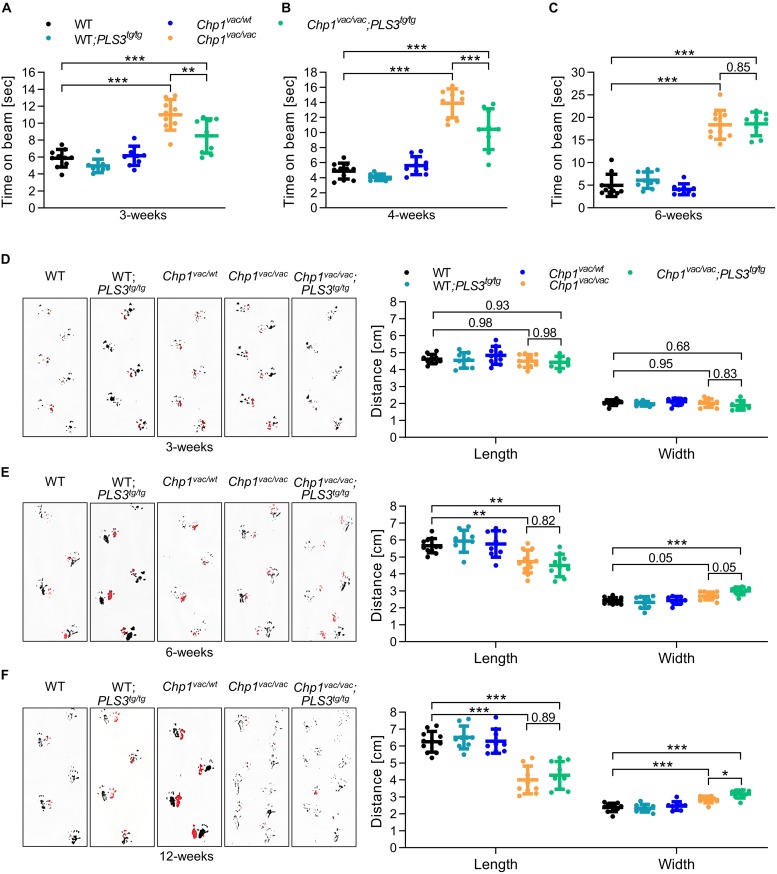
PLS3 OE delays the ataxia phenotype in *vacillator* mice. Beam-walking assay and footprint pattern analysis of WT, WT-*PLS3*^tg/tg^, *Chp1*^vac/wt^, *Chp1*^vac/vac^ and *Chp1^vac/vac^;PLS3^tg/tg^* mice during disease progression. **(A–C)** The time to cross the beam was measured in 3-, 4-, and 6-week-old mice. The performance of *Chp1*^vac/vac^ mice was significantly reduced compared to WT mice. PLS3 OE improved the performance of *Chp1^vac/vac^;PLS3^tg/tg^* in 3- and 4-week-old but not at 6-week-old mice (*N* = 8–11). **(D–F)** Representative footprint patterns and quantifications in 3-, 6-, and 12-week-old mice. *Chp1*^vac/vac^ and *Chp1^vac/vac^;PLS3^tg/tg^* mice displayed a progressive ataxic gait starting at 6-weeks of age (*N* = 7–11). Rear paws (black) and front paws (red). ^∗^*p* < 0.05, ^∗∗∗^*p* < 0.01, ^∗∗∗^*p* < 0.001, one-way ANOVA and Holm–Sidak’s multiple comparisons test. Error bars represent SD.

The beam-walking assay performance (Stanley et al., 2005) was estimated by recording the time the mouse needed to cross the beam. Remarkably, *Chp1*^vac/vac^ mice (11.00 s) showed a strongly reduced performance already at 3-weeks of age compared to WT mice (5.86 s) (Figure 2A and Supplementary Table S1), confirming the sensitivity of the test. While the performance of *Chp1*^vac/vac^ mice was significantly improved upon PLS3 OE at an early disease stage of 3- and 4-weeks of age (3-weeks: 8.50 s; 4-weeks: 10.45 s), compared to *Chp1*^vac/vac^ mice (3-weeks: 11.00 s; 4-weeks: 13.88 s) (Figures 2A,B, Supplementary Table S1 and Movies), no difference at a later disease stage of 6 weeks was detectable (Figure 2C), suggesting that the PLS3 OE ameliorates only early neuronal processes and circuits disturbed by CHP1 depletion, but has no ameliorating effect at later disease stages.

Additionally, to record motoric coordination we performed a footprint pattern analysis (Tsai et al., 2012), since an ataxic gait has characteristic features including decreased stride length and increased stride width. Despite *Chp1*^vac/vac^ mice display already severe balancing problems at 3- and 4-weeks of age, no significant differences in the footprint pattern analysis were observed at this early time point (Figure 2D). Instead, 6-week-old *Chp1*^vac/vac^ mice showed a prominent ataxic gait, with reduced stride length and increased stride width as compared to WT mice, which became even more pronounced in 8- and 12-week-old mice (Figures 2E,F and Supplementary Figure 1A). In contrast to the beam-walking test, no overt difference was observed between *Chp1*^vac/vac^ and *Chp1^vac/vac^;PLS3^tg/tg^* mice at an early disease stage, while at 12-weeks of age, PLS3 OE seemed to slightly worsen the stride width.

Moreover, a footprint overlap analysis (Carter et al., 1999) did not show any differences among genotypes at 3- and 6-weeks of age. At 12-weeks of age, PLS3 OE showed a worsening effect in *vacillator* mice (Supplementary Figure 1B). This data is consistent with the increased width stride observed in the same group of mice (Figure 2F).

Overall, the outcome of our motor-balancing and coordination tests suggest on one hand, that balancing is early affected in the *vacillator* mice, as very well reflected by the beam-walking test at 3 and 4-weeks of age, but significantly delayed by PLS3 overexpression. On the other hand, while the footprint test very well captures the characteristic motor coordination impairment of the *vacillator* mice at 6-weeks of age and later, these defects are not rescued by PLS3 OE. Rather paradoxically, at a late stage, PLS3 OE seems to mildly worsen the phenotype. This could be either due to very advanced Purkinje neurons degeneration or due to the specific affection of neuronal circuits responsible for balancing and gait motor abilities.

### PLS3 OE Delays Purkinje Neuron Degeneration

The *Chp1* mutant mouse model was originally reported to develop cerebellar ataxia caused by Purkinje neuron degeneration in the cerebellum. In detail, it has been shown that axon degeneration precedes Purkinje neuron loss, suggesting a dying-back mechanism, where axonal degeneration precedes cell body death. In congruence, first axonal signs of Purkinje neuron degeneration were already prominent at P15 (Liu et al., 2013). Since we demonstrated a beneficial effect of PLS3 OE on motor balance and coordination at an early disease stage of 3-weeks, we speculated that PLS3 OE may delay Purkinje neuron degeneration, thereby ameliorating the disease phenotype.

**FIGURE 3:**
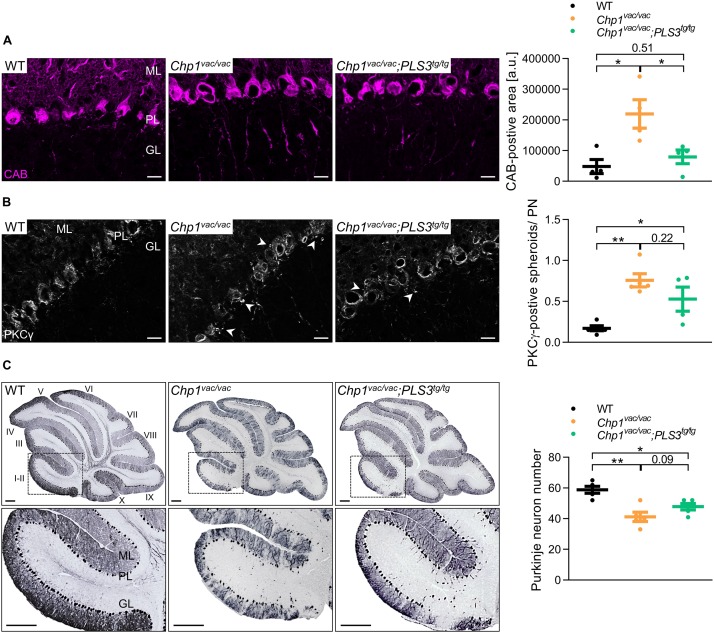
PLS3 OE ameliorates Purkinje neuron degeneration. **(A,B)** Representative pictures and quantification of sagittal cryo-sections of cerebellum from WT, *Chp1*^vac/vac^ and *Chp1^vac/vac^;PLS3^tg/tg^* in 3-week-old mice. **(A)** *Chp1*^vac/vac^ Purkinje neurons presented axonal swelling and hypertrophy. Axonal swellings, measured by axonal calbindin-D28k (CAB)-positive area in cerebellar lobules I-II (see inset in **C**), were significantly reduced in *Chp1^vac/vac^;PLS3^tg/tg^* mice. CAB (magenta, for Purkinje neuron) (scale bar: 20 μm) (*N* = 4). **(B)** *Chp1*^vac/vac^ mice presented PKCγ-positive enlarged spheroids (arrowheads) beneath the Purkinje cell and lower molecular layer representing axon collateral degeneration. PLS3 OE led to a trend toward reduced PKCγ-positive spheroids per Purkinje neuron (PN). Spheroids were quantified in cerebellar lobules I-II, PKCγ (white) (scale bar: 20 μm) (*N* = 4). **(C)** Representative pictures of sagittal paraffin sections of cerebellum of WT, *Chp1*^vac/vac^ and *Chp1^vac/vac^;PLS3^tg/tg^* mice at 16-weeks of age. Quantification of Purkinje neuron number in cerebellar lobules I-II demonstrated early Purkinje neuron as well as marked axon degeneration in cerebella from *Chp1*^vac/vac^ mice. *Chp1^vac/vac^;PLS3^tg/tg^* mice presented a trend toward increased Purkinje neuron number compared to *Chp1*^vac/vac^ mice (*N* = 5). Purkinje neurons were immunostained with CAB (scale bar: 200 μm). Roman numerals indicate cerebellar lobules. ML: molecular layer, PL: Purkinje cell layer, GL: granule cell layer. ^∗^*p* < 0.05, ^∗∗^*p* < 0.01, one-way ANOVA and Holm–Sidak’s multiple comparisons test. Error bars represent SD.

To test this hypothesis, we performed immunohistochemistry of sagittal brain sections from 3-week-old mice using the Purkinje neuron markers calbindin-D28k (CAB) and protein kinase C gamma (PKCγ). In line with a previous study (Liu et al., 2013), *Chp1*^vac/vac^ mice displayed a widespread hypertrophy of Purkinje neuron axons which was not present in WT (Figure 3A). Interestingly, upon PLS3 OE the extent of Purkinje axon hypertrophy and axonal swellings appeared to be reduced. In order to quantify the axon hypertrophy, we assessed the CAB-positive area. Thereby, we could not only show that *Chp1*^vac/vac^ mice exhibit a strongly increased CAB-positive area, confirming the axon hypertrophy, but also show that upon PLS3 OE in *Chp1*^vac/vac^ mice the CAB-positive area was reduced, implying diminished axon hypertrophy upon PLS3 OE.

Abnormal PKCγ-positive focal swellings/spheroids, representing axon collaterals, have previously been described as an early hallmark of the onset of Purkinje neuron degeneration in the *vacillator* mice (Liu et al., 2013). Quantification of the PKCγ-positive spheroids per Purkinje neuron further demonstrated that *Chp1*^vac/vac^ mice displayed significantly elevated PKCγ-positive spheroids than WT mice (Figure 3B). Moreover, although not significant, *Chp1^vac/vac^;PLS3^tg/tg^* mice presented a strong trend toward a diminished number of PKCγ-positive spheroids compared to *Chp1*^vac/vac^ mice (Figure 3B), suggesting delayed axonal impairment of Purkinje neurons.

Although we only observed a beneficial effect of PLS3 OE on motor performance at an early disease stage, we also aimed to investigate the effect of PLS3 on Purkinje neuron morphology at a later symptomatic disease stage. Since previous studies of the *vacillator* mouse model reported that Purkinje neuron loss was apparent only at 4 months (Liu et al., 2013), we quantified the Purkinje neuron loss and the effect of PLS3 OE at this time point. Consistent with previous results, *Chp1*^vac/vac^ mice displayed a marked reduction of Purkinje neurons as well as a complete loss of Purkinje neuron axons compared to WT mice (Figure 3C), while no difference was observed at 5-weeks of age (Supplementary Figure S2). Although Purkinje neuron axons were absent, PLS3 OE seems to have an ameliorating effect on these cells, as a consistent trend of an increased Purkinje neuron number was observed in cerebella of *Chp1^vac/vac^;PLS3^tg/tg^* mice compared to *Chp1*^vac/vac^ mice at 4 months (Figure 3C).

Taken together, we showed that PLS3 OE delays axonal degeneration of Purkinje neurons at 3-weeks of age, eventually slowing down Purkinje neuron loss. Nevertheless, Purkinje neuron death eventually occurs also in *Chp1^vac/vac^;PLS3^tg/tg^* mice. Thus, the delayed axon hypertrophy in the *Chp1^vac/vac^;PLS3^tg/tg^* mice, has an ameliorating effect on the disturbed coordination/balancing ability at early disease stages, as shown by an improved performance in the beam-walking test.

### PLS3 OE Improves Membrane Targeting of NHE1 in *Vacillator* Mice

Next, we wanted to elucidate the mechanism by which PLS3 OE delays axonal hypertrophy of Purkinje neurons. In previous studies, it has been proposed that CHP1 reduction results in decreased NHE1 glycosylation, leading to impaired membrane targeting, resulting in disturbed pH homeostasis, likely causing Purkinje neuron death (Liu et al., 2013; Mendoza-Ferreira et al., 2018).

**FIGURE 4:**
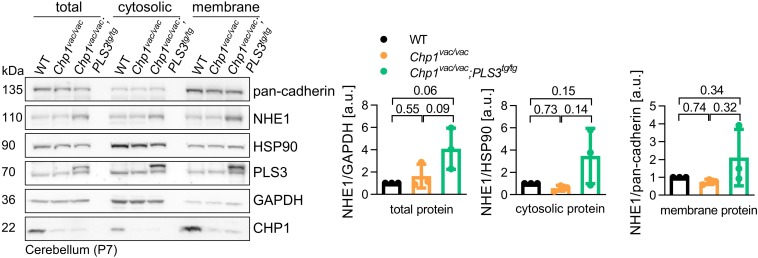
Analysis of PLS3 OE on membrane targeting of NHE1 in *vacillator* mice. Representative Western blot and quantification of subcellular fractionation of cerebella from WT, *Chp1*^vac/vac^ and *Chp1^vac/vac^;PLS3^tg/tg^* mice at P7. NHE1 level was slightly decreased in the membrane fraction of *Chp1*^vac/vac^ cerebellum compared to WT. Upon PLS3 OE, NHE1 was enriched in the cerebellum membrane fraction of *Chp1^vac/vac^;PLS3^tg/tg^* compared to *Chp1*^vac/vac^ mice (*N* = 3). Pan-cadherin and EGFR: membrane markers, HSP90 and GAPDH: cytosolic markers. One-way ANOVA and Holm–Sidak’s multiple comparisons test. Error bars represent SD.

To test whether PLS3 OE plays a role during this process and improves NHE1 membrane targeting, we performed subcellular fractionation of cerebella from WT, *Chp1*^vac/vac^ and *Chp1^vac/vac^;PLS3^tg/tg^* mice at P7. This early time point was chosen since subcellular fractionation from older brains is impeded, due to high lipid concentration. However, compared to previous finding (Liu et al., 2013), we found only slightly reduced NHE1 membrane targeting in *Chp1*^vac/vac^ cerebellum compared to WT. Nevertheless, PLS3 OE strongly increased NHE1 levels in the membrane fractions of *Chp1*^vac/vac^ cerebellum (Figure 4). Most strikingly, PLS3 OE led to a robust trend toward increased NHE1 levels in total lysate, cytoplasmic and membrane fractions (Figure 4).

Taken together, here, we demonstrated that PLS3 OE not only increases total NHE1 levels but seems to have also a positive effect on the membrane localization in the cerebellum, which is specifically sensitive toward CHP1 depletion. These results suggests that PLS3 OE most likely delays the early ataxia disease progression and Purkinje neuron pathology by increasing and stabilizing NHE1 membrane localization and thereby eventually ameliorating impaired pH homeostasis.

## Discussion

The main findings of this study are: (1) PLS3 OE ameliorates the ataxic phenotype of *Chp1* mutant mice at an early disease stage at 3- and 4-weeks of age but not at 6-weeks of age when the phenotype is more pronounced; (2) PLS3 OE ameliorates axon hypertrophy and axonal swellings in Purkinje neurons at 3-weeks of age; and (3) PLS3 OE appears to increase NHE1 levels and membrane localization in *Chp1*^vac/vac^ cerebellum, thereby most likely delaying Purkinje neuron axon pathology and eventually slowing down neurodegeneration.

It has been previously proposed that neurodegenerative disorders including SMA, ALS, Parkinson‘s disease, and hereditary spastic paraplegias share common pathogenic pathways such as endocytic defects, Ca^2+^ misregulation and defects in actin dynamics (Hosseinibarkooie et al., 2016; Schreij et al., 2016; Hensel and Claus, 2018). One factor improving these common pathways is PLS3, highlighting the potential of PLS3 OE as a cross-disease modifier (Hosseinibarkooie et al., 2016). Interestingly, in previous mass spectrometry analysis, we could link PLS3 to numerous proteins associated with various neurodegenerative diseases (Hosseinibarkooie, 2016). Since PLS3 has been shown to directly interact with CHP1 (Janzen et al., 2018), in this study we investigated whether PLS3 is a cross-disease modifier for ataxia caused by *Chp1* mutation in mice. Interestingly, we show that PLS3 OE delays the ataxic phenotype in an ARCA mouse model, i.e., the *vacillator* mouse model, caused by homozygous loss-of-function mutation in *Chp1*. Previously it has been corroborated that CHP1 is an essential cofactor for the glycosylation of NHE1, which is crucial for the membrane targeting of NHE1. Consequently, loss-of-function mutations and diminished levels of CHP1 lead to altered NHE1 maturation, causing defective pH homeostasis in Purkinje neurons, thereby leading to axon degeneration and subsequent cell death and eventually to ataxia (Liu et al., 2013; Mendoza-Ferreira et al., 2018). Congruently, *Nhe1* KO mice display a similar ataxic phenotype as *Chp1* mutant mice (Cox et al., 1997; Bell et al., 1999), suggesting that NHE1 is downstream of CHP1. This work not only demonstrated that PLS3 OE delays the progression of the ataxic gait, as assessed by the beam-walking assay at an early disease stage, but also show that PLS3 OE delays axonal impairment of Purkinje neurons. Since disturbed NHE1 localization and pH homeostasis have been associated with the phenotype of *Chp1* mutant mice, we speculated that PLS3 OE could affect NHE1 localization. Indeed, PLS3 OE seems to have a beneficial effect on NHE1 levels and membrane targeting. Although NHE1 levels in *Chp1^vac/vac^;PLS3^tg/tg^* cerebella were strongly increased in total, cytosolic and membrane fractions compared to *Chp1*^vac/vac^ cerebella, the difference was not significant, due to overt variations among mice. Nevertheless, the trend toward increased NHE1 targeting upon PLS3 OE was very consistent, thereby implying that PLS3 may ameliorate the progression of the ataxic phenotype through this mechanism.

However, how PLS3 facilitates NHE1 membrane targeting remains elusive. We developed the following hypotheses on how PLS3 OE might slow down Purkinje neuron degeneration: (1) since PLS3 interacts with CHP1, it is likely that both proteins may function together in common pathways, stabilize each other, or affect each other’s localization. Hence, PLS3 OE might stabilize the remaining CHP1 protein and consequently increase glycosylation and targeting of NHE1. However, the experiment analyzing the dependence of PLS3 and CHP1 on each other’s localization in *Chp1*^vac/vac^ and *Pls3*^ko/ko^ MEFs, did not reveal any direct connection (data not shown). (2) NHE1 has been shown to directly interact with the actin binding proteins ezrin, radixin, and moesin (ERM) and acts as an anchor for actin filaments (Denker et al., 2000). PLS3 is an actin-binding and bundling protein, whose OE has been shown to increase F-actin levels (Hosseinibarkooie et al., 2016). Thus, PLS3 may improve NHE1 targeting by increasing or stabilizing F-actin filaments and thereby eventually promoting the anchoring of the NHE1-ERM complex to the membrane. (3) PLS3 OE may ameliorate the early disease pathology also through an NHE1-independent mechanism. Neurons are highly polarized cells, that undergo complex morphological rearrangements to assemble into neuronal circuits and propagate signals (Tahirovic and Bradke, 2009). Therefore, the cytoskeleton is essential for neuronal polarization, while axon specification, in particular, requires local actin dynamics and stabilization of microtubules (Witte and Bradke, 2008). Consequently, PLS3 OE may slow down the observed Purkinje axon hypertrophy and eventually neurodegeneration, through an overall axonal stabilization by increasing F-actin levels.

Importantly, both PLS3 OE and CHP1 reduction have previously been shown to be protective modifiers of the neurodegenerative disorder SMA. Hereby, endocytosis has been elucidated as an underlying protective mechanism. While PLS3 OE improved the impaired endocytosis by increasing F-actin levels, CHP1 reduction increased calcineurin activity, thus restoring dynamin-1 phosphorylation and eventually endocytosis (Oprea et al., 2008; Hosseinibarkooie et al., 2016; Janzen et al., 2018). Importantly, in the context of SMA, about 50% CHP1 reduction was beneficial, whereas in case of ataxia, CHP1 levels were dramatically reduced, causing impaired NHE1 maturation and imbalance of pH homeostasis (Liu et al., 2013; Janzen et al., 2018). Thus, we speculate that the effects of CHP1 reduction on ameliorating SMA pathology or causing ataxia mostly rely on different functions and dosages of CHP1.

It remains hitherto unknown, how exactly PLS3 OE delays the ataxia progression at an early stage. Further studies such as addressing the discussed hypotheses are required to elucidate the underlying molecular mechanisms. Nevertheless, this study highlights the involvement of PLS3 OE as a cross-disease modifier for neurodegenerative diseases, including SMA and *CHP1*-associated ataxia.

## Data Availability

The raw data supporting the conclusions of this manuscript will be made available by the authors, without undue reservation, to any qualified researcher.

## Ethics Statement

The animal study was reviewed and approved by the LANUV NRW (reference number: 84-02.04.2014.A242).

## Author Contributions

EJ and BW designed the project. EJ carried out all the experiments with the help of NM-F, LW, AD, KH, SH, and MK and wrote the manuscript with the help of all co-authors.

## Conflict of Interest Statement

BW, EJ, NM-F, and SH hold an EP 17172826.4 filed May 24, 2017 entitled “Calcineurin B Homologous Protein 1 Inhibitors and Therapeutic and Non-Therapeutic Uses Thereof.” The remaining authors declare that the research was conducted in the absence of any commercial or financial relationships that could be construed as a potential conflict of interest.
