# Supplementary material for: PLS3 Overexpression Delays Ataxia in Chp1 Mutant Mice
Source: Front Neurosci. 2019 Sep 19;13:993. doi: 10.3389/fnins.2019.00993 (PMC6761326; doi:10.3389/fnins.2019.00993)
Supplement: Supplementary file 1 [file Data_Sheet_1.pdf]

Supplementary Table 1. Raw data for beam-walking and foot print analyses. In tables: N indicates the number of mice per genotype. Data is presented as mean value ± SD. Data is associated column-wise to the above indicated figures.

Genotype	Figure 2A; 3-weeks		Figure 2B; 4-weeks		Figure 2C; 6-weeks	
	N	Mean [sec] ± SD	N	Mean [sec] ± SD	N	Mean [sec] ± SD
WT	10	5.86 ± 1.03	11	4.87 ± 1.05	10	4.96 ± 2.46
WT; *PLS3*$^{tg/tg}$	8	4.96 ± 0.78	9	4.06 ± 0.44	9	6.12 ± 1.81
Chp1$^{vac/wt}$	9	6.15 ± 0.78	9	5.97 ± 1.20	9	4.11 ± 1.20
Chp1$^{vac/vac}$	9	11.00 ± 1.81	9	13.88 ± 1.92	10	18.38 ± 3.21
Chp1$^{vac/vac}$; *PLS3*$^{tg/tg}$	9	8.50 ± 2.03	9	10.45 ± 2.71	8	18.6 ± 2.62

Genotype	Figure 2D; 3-weeks			Figure 2E; 6-weeks			Figure 2F; 12-weeks		
	N	Mean length ± SD	Mean width ± SD	N	Mean length ± SD	Mean width ± SD	N	Mean length ± SD	Mean width ± SD
WT	10	4.62 ± 0.28	2.05 ± 0.17	12	5.66 ± 0.42	2.42 ± 0.18	11	6.26 ± 0.61	2.38 ± 0.25
WT; *PLS3*$^{tg/tg}$	8	4.54 ± 0.45	1.98 ± 0.14	9	5.93 ± 0.65	2.32 ± 0.35	9	6.51 ± 0.67	2.33 ± 0.23
Chp1$^{vac/wt}$	9	4.84 ± 0.53	2.11 ± 0.21	8	5.77 ± 0.78	2.44 ± 0.23	9	6.29 ± 0.71	2.46 ± 0.26
Chp1$^{vac/vac}$	9	4.51 ± 0.38	2.02 ± 0.24	11	4.73 ± 0.67	2.70 ± 0.23	10	4.00 ± 0.82	2.85 ± 0.20
Chp1$^{vac/vac}$; *PLS3*$^{tg/tg}$	7	4.43 ± 0.36	1.89 ± 0.27	9	4.5 ± 0.67	3.00 ± 0.22	9	4.27 ± 0.82	3.17 ± 0.24

Genotype	Data not shown; 4-weeks			Supplementary Figure 1A; 8-weeks		
	N	Mean length ± SD	Mean width ± SD	N	Mean length ± SD	Mean width ± SD
WT	11	5.13 ± 0.26	2.29 ± 0.22	10	6.29 ± 0.58	2.29 ± 0.21
WT; *PLS3*$^{tg/tg}$	9	4.89 ± 0.62	2.14 ± 0.31	9	6.19 ± 0.63	2.28 ± 0.34
Chp1$^{vac/wt}$	9	5.38 ± 0.27	2.24 ± 0.17	9	5.94 ± 0.77	2.49 ± 0.32
Chp1$^{vac/vac}$	8	4.81 ± 0.35	2.32 ± 0.33	10	4.58 ± 0.67	2.88 ± 0.22
Chp1$^{vac/vac}$; *PLS3*$^{tg/tg}$	7	4.61 ± 0.43	2.43 ± 0.29	9	4.52 ± 0.89	3.03 ± 0.24

Supplementary Figure 1

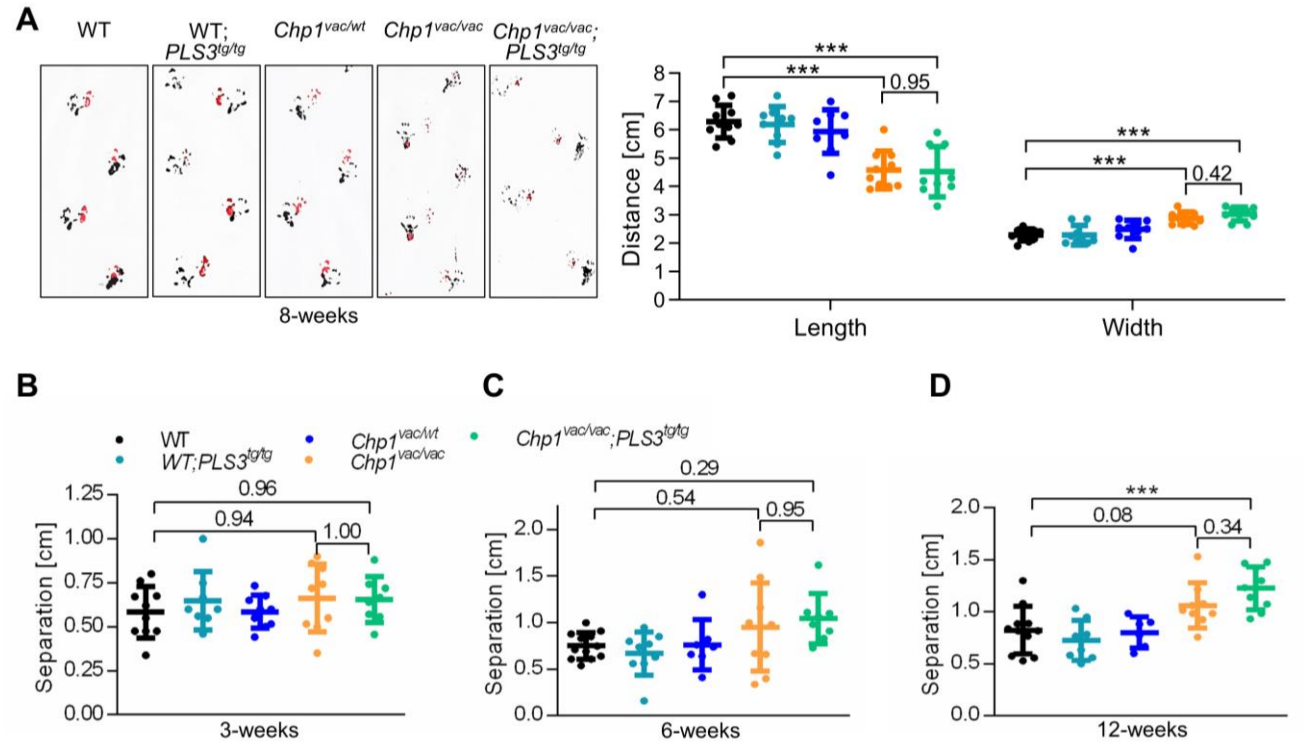

Supplementary Figure 1: (A) Representative footprint patterns and quantifications of 8-week-old mice (N=7-11). Rear paws (black) and front paws (red). **(B-C)** Step overlap analysis of 3-week-, 6-week- and 12-week-old mice. ***p <0.001, one-way ANOVA and Holm-Sidak's multiple comparisons test. Error bars represent SD.

Supplementary Figure 2

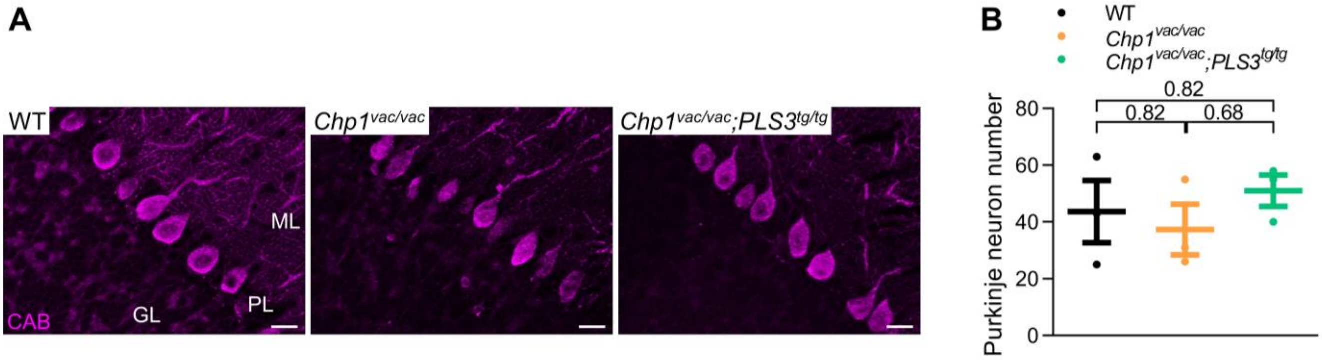

Supplementary Figure 2: (A) Representative pictures of sagittal paraffin sections of cerebellum from WT, *Chp1*$^{vac/vac}$ and *Chp1*$^{vac/vac}$*;PLS3*$^{tg/tg}$ in 5-week-old mice. CAB (magenta, for Purkinje neuron), ML: molecular layer, PL: Purkinje cell layer, GL: granule cell layer (scale bar: 20 µm). **(B)** Quantification of Purkinje neuron number in cerebellar lobules I-II does not reveal any difference in Purkinje neuron number (N=3). One-way ANOVA and Holm-Sidak's multiple comparisons test. Error bars represent SD.

Supplementary Videos

Beam-walking test in **(1)** wildtype mouse, **(2)** Chp1$^{vac/vac}$ mouse, **(3)** Chp1$^{vac/vac}$; PLS3$^{tg/tg}$ mouse
